# Quantum-Centric Computational Study of Methylene Singlet and Triplet States

**DOI:** 10.1021/acs.jctc.5c00075

**Published:** 2025-05-13

**Authors:** Ieva Liepuoniute, Kirstin D. Doney, Javier Robledo Moreno, Joshua A. Job, William S. Friend, Gavin O. Jones

**Affiliations:** † IBM Research—Almaden, IBM Quantum, 650 Harry Road, San Jose, California 95120, United States; ‡ Lockheed Martin, 3251 Hanover Street, Palo Alto, California 94304, United States; § T. J. Watson Research Center, IBM Quantum, Yorktown Heights, New York 10598, United States

## Abstract

This study involves quantum simulations of the dissociation of the ground-state triplet and first excited singlet states of the CH_2_ molecule (methylene), which are relevant for interstellar and combustion chemistry. These were modeled as (6e, 23o) systems using 52 qubits on a quantum processor by applying the sample-based quantum diagonalization (SQD) method within a quantum-centric supercomputing framework. We evaluated the ability of SQD to provide accurate results of the singlet-triplet gap in comparison to selected configuration interaction (SCI) calculations and experimental values. To our knowledge, this is the first study of an open-shell system (the CH_2_ triplet) using SQD. To obtain accurate energy values, we implemented post-SQD orbital optimization and employed a warm-start approach using previously converged states. The results for the singlet state dissociation were highly accurate, differing by only a few milli-Hartrees from the SCI reference values. Similarly, the SQD-calculated singlet-triplet energy gap aligned well with both experimental and SCI values, underscoring the method’s capability to capture key features of CH_2_ chemistry. However, the triplet state exhibited greater variability, likely due to differences in bit-string handling within the SQD method for open- versus closed-shell systems and the inherently complex wavefunction character of the triplet state. These findings highlight the strengths and limitations of SQD for modeling open-shell systems while laying a foundation for its application in large-scale electronic structure studies using quantum algorithms.

## Graphical abstract

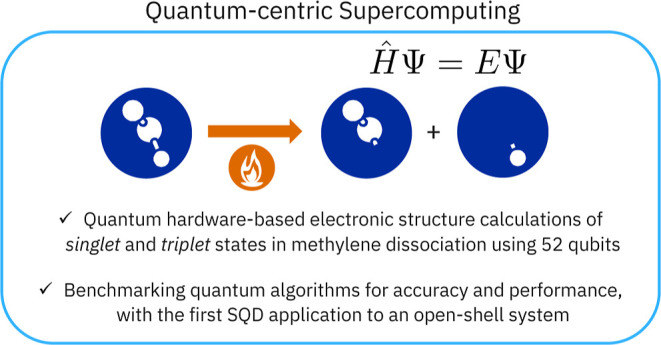

## 1 Introduction

Accurate electronic structure calculations are essential for simulating molecules as they provide detailed descriptions of molecular energetics, reaction pathways, and spectroscopic properties.^1–3^ These quantum chemical calculations enable precise predictions of molecular behavior and spectroscopic properties, proving invaluable in understanding transient, radical, or toxic molecules that are difficult to measure experimentally^4–6^ . Classical quantum chemical calculation methods, such as coupled-cluster theory (CC) or density functional theory (DFT), while fundamental, can become computationally expensive for large, polyatomic molecules of chemical interest or may lack the necessary accuracy.^7,8^ This is particularly true when addressing the highly entangled nature of open-shell radicals. Recent advances in quantum computing present new opportunities for effectively modeling these intricate systems.^9–12^ Most notably, recent studies have demonstrated that electronic structure calculations of chemical relevance for multi-atom and heavy atom molecules,^9^ as well as supramolecular systems^13^ is now feasible using the sample-based quantum diagonalization (SQD) technique–a generalization of quantum selected configuration interaction^14^–within a quantum-centric supercomputing (QCSC) framework.^9,13–17^ The SQD technique has been successfully applied to electronic structure problems, including an iron–sulfur cluster (77 qubits) and calculations of hydrophobic interactions in a methane dimer (up to 54 qubits);^9,13^ it has also been employed in combination with Krylov methods to study impurity models^18^ and integrated with Quantum Monte Carlo methods;^19^ but its application to open-shell systems has not yet been explored.

Methylene (CH_2_) is a molecule of considerable importance in both interstellar and combustion chemistry.^20–23^ As the prototypical carbene and the smallest polyatomic free radical, it serves as a benchmark for evaluating the performance of theoretical and computational methodologies.^24,25^ Computationally, CH_2_ is distinguished by its triplet radical ground state (X̃B13) and the pronounced multireference character of its lowest singlet excited state (ãA11) , making it an ideal system for testing and validating advanced electronic structure methods.^24,26–29^ The geometry and the equilibrium singlet-triplet energy gap (*T*_e_) have been confirmed by extensive experimental and computational studies.^24,26–33^ For the two lowest states of CH_2_, the equilibrium geometry is *C*_2v_. The ground state is an open-shell triplet state, X̃B13 , which has a C–H bond length of ca. 1.09 Å and a H–C–H angle of ca. 135.5°,^33^ with the electronic configuration ${\left(1a_{1}\right)}^{2}{\left(2a_{1}\right)}^{2}{\left(1b_{2}\right)}^{2}\left(3a_{1}\right)\left(1b_{1}\right)$ . Conversely, the first excited state is a closed-shell singlet state, ãA11 , which has a C–H bond length of 1.11 Å and a H–C–H angle of ca. 102.4°,^32^ with two important electronic configurations: ${\left(1a_{1}\right)}^{2}{\left(2a_{1}\right)}^{2}{\left(1b_{2}\right)}^{2}{\left(3a_{1}\right)}^{2}$ and ${\left(1a_{1}\right)}^{2}{\left(2a_{1}\right)}^{2}{\left(1b_{2}\right)}^{2}{\left(1b_{1}\right)}^{2}$ . The orbital occupation of the last two electrons defines the specific electronic state of methylene, as depicted in Figure 1. At the lowest point in the respective potential energy surfaces (PESs), the energy gap between the two states *T*_e_ is 14 mE_h_ (i.e., 3159 cm^–1^ or 9.03 kcal/mol).^28,34^

**Figure 1:**
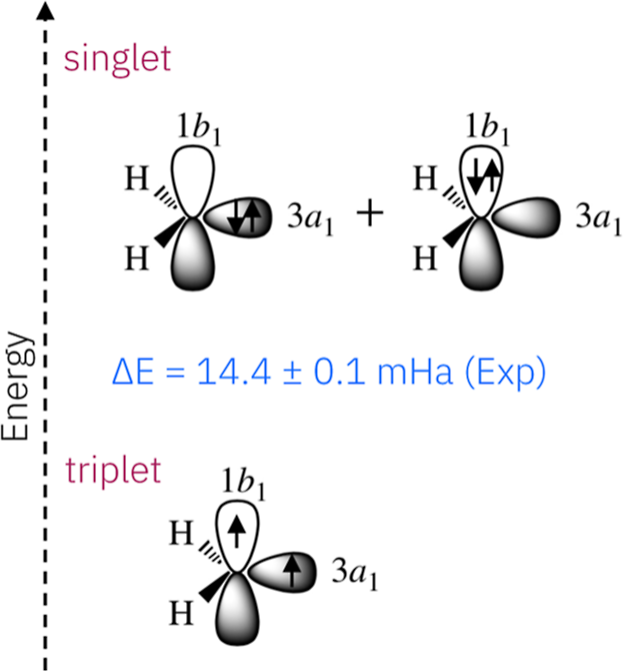
Highest occupied molecular orbitals of methylene, along with the corresponding electronic configurations for the ground-state triplet and the first excited-state singlet. The experimentally determined singlet-triplet energy gap is ca. 14 mE_h_ at *T*_e_.

Very accurate classical calculations approaching the complete basis set limit are needed to reproduce the experimental value of the singlet-triplet gap. Despite the efforts of various computational methods, significant discrepancies persist. The challenges encountered by DFT and other methods in studying the CH_2_ singlet-triplet gap are systematically documented in ref 35 The Hartree–Fock (HF) method, for example, grossly overestimates this gap due to the necessity of including a second configuration in the reference description of the ãA11 state. This overestimation is also evident in the MP2 method, which relies on an insufficient single-configuration (ref 36) Furthermore, the gap shows a pronounced basis set effect, decreasing from 21.7 mE_h_ with a double-zeta polarized basis to 18 mE_h_ with a triple-zeta polarized 2 (TZ2P) basis, and further to 17.4 mE_h_ with a triple-zeta polarized 3 (TZ3P) basis. All ab initio methods exhibit this basis set effect on the energy difference, with predictions ranging from 14 to 18 mE_h_ when using at least a TZP basis set. DFT methods yield similar results. The most accurate calculation to date, utilizing the MRCISD/aug-cc-pV6Z method, predicts a gap that differs from the experimental results by 0.2 mE_h_.^37^

The methylene ground state, a triplet, exemplifies an open-shell system characterized by unpaired electrons or partially filled orbitals. Other prominent examples include transition-metal complexes, low-lying excited states, radicals, and diradicals. Such systems present substantial challenges to classical computational methods due to their complex electronic properties. In general, open-shell wavefunctions frequently become multiconfigurational, particularly in the presence of electronic degeneracies such as partially broken bonds, charge-transfer states, or spin-adapted configurations.^38,39^ Single-determinant methods like HF and DFT are inherently limited in these cases, as they fail to capture the multiple leading configurations required for an accurate description.^7,38–41^ Additionally, the accurate treatment of these systems is complicated by several challenges, such as spin contamination and symmetry breaking in the reference wavefunction, instabilities and near-singularities in the Hartree-Fock solution, strong electron correlation effects, and intricate features of adiabatic potential energy surfaces.^42^ For example, excited-state spin contamination can lead to errors as large as 1–3 eV even for simple systems like *N*_2_^+^.^43^ While many-body methods built upon HF, such as coupled-cluster theories, address some limitations by incorporating electron correlation, their accuracy is insufficient for strongly correlated systems.^38,41^ Achieving true balance remains feasible only at the computationally prohibitive level of a full configuration interaction (FCI). To date, computational resource limitations have restricted accurate FCI calculations to relatively small systems. Notably, the largest FCI calculation to date, involving 1.31 trillion determinants, successfully computed the exact energy of C_3_H_8_ within a (26e, 23o) active space using the STO-3G basis set.^44^ Multireference methods, though more accurate for open-shell systems, remain computationally demanding, underscoring the need for advanced approaches to address the complexities of open-shell species. Emerging quantum computing methods offer a promising avenue to address these challenges, leveraging quantum hardware to simulate open-shell systems efficiently by naturally incorporating multiconfigurational effects and reducing computational overhead in strongly correlated regimes.^45^

In this study, we performed SQD calculations on the singlet and triplet states of CH_2_ using a correlation-consistent cc-pVDZ basis set, encompassing 6 electrons across 23 orbitals. This work represents the first open-shell analysis of molecular dissociation using SQD with a realistic correlation-consistent basis and the first study of excited states on different U(1) symmetry sectors. Additionally, this is the first study of a quantum phase transition that results from a level crossing using SQD. To assess the accuracy of the results, we compared them with SCI calculations as implemented in PySCF^46,47^ and experimental energy values, providing insight into the capabilities of the SQD algorithm for both closed- and open-shell systems.

The structure of this work is as follows: first, we describe the methods employed with a focus on large-scale noisy quantum hardware simulations and the associated error mitigation techniques. Next, we present and discuss the electronic energy results for the CH_2_ triplet and singlet states and the calculation of the singlet-triplet gap using the SQD technique by simulating the C–H bond stretching.

## 2 Methods

### 2.1 Quantum Algorithms

#### 2.1.1 Sample-Based Quantum Diagonalization

SQD^9,14,17^ is a variational approach for the search of eigenstates of many-body systems. The wavefunction ansatz is based on the expansion of a general many-body state in a subset S (of polynomial size in the number of electrons and orbitals) of the basis of single-particle electronic configurations

1 $$|\psi \rangle =\sum_{x\in S}\psi_{x}|x\rangle$$

where $x\in {\left\{0,1\right\}}^{2N_{orb}}$ are bit-strings whose length is twice the number of spatial orbitals. The first and second halves of each bit-string represent the occupancy of the spin-up and spin-down orbitals, respectively. |**x**⟩ represents a Slater determinant (or electronic configuration) of the form

2 $$|x\rangle =\prod_{p,\sigma }{\left({â}_{p\sigma }^{†}\right)}^{x_{p\sigma }}|0\rangle$$

where *p* = 1, 2, ..., *N*_orb_, and σ ∈ {↑, ↓}. The coefficients of expansion ψ_**x**_ are defined by a lookup table. Provided that |S| grows polynomially with system size, their optimal values can be obtained by the diagonalization of the many-body Hamiltonian projected into the subspace S

3 $${Ĥ}_{S}={\mathrm{P̂}}_{S}Ĥ{\mathrm{P̂}}_{S}$$

where the projector ${\mathrm{P̂}}_{S}$ is

4 $${\mathrm{P̂}}_{S}=\sum_{x\in S}|x\rangle \langle x|$$

The size and constituents of S determine the accuracy of |Ψ⟩ to represent the target eigenstate. Classical heuristics exist to search the set of relevant configurations to include in S , which fall under the general umbrella of SCI methods.^48–51^ Recent studies suggest that a quantum circuit Ψ can also produce accurate statistical models $p\left(x\right)={\left|\langle x|\Psi \right|}^{2}$ to produce samples that belong to S .^9^ In this second form, quantum circuit Ψ is prepared and measured in the computational basis. Under the Jordan–Wigner^52^ encoding, the sampled bit-strings are identified with the electronic configurations x∈S . Since the interacting electron problem preserves the total number of electrons and the number of spin-up and spin-down electrons separately, the Hamming weight of each half of any sampled bit-string must be equal to the number of spin-up and spin-down electrons. Therefore, circuits that are particle-number preserving are considered (see Section 2.1.5).

#### 2.1.2 Self-Consistent Configuration Recovery

Quantum noise perturbs the distribution *p*(**x**), leading to a modified distribution *p̂*(*x*), which impacts the accuracy of the variational ansatz representation. Consequently, the quantum device produces noisy samples that may break the particle-number and *z* – components of spin symmetries. Reference 9 introduces a self-consistent procedure to restore particle-number conservation using information from the diagonal of the one-body reduced density matrix $\left\langle {â}_{p\sigma }^{†}{â}_{p\sigma }\right\rangle$ . The value of *n*_*p*σ_ is not assumed to be known a priori but is computed and refined self-consistently, as depicted in panel (b) of Figure 2. The quantum processor produces a set of noisy measurement outcomes X̃ , which are processed following a self-consistent iterative procedure:

1. **Setup**: From the set of electronic configurations in X̃ , the ones with the wrong particle number and *z*-component of the spin are ignored, yielding a set of physical electronic configurations. The resulting configurations define the subspace S and are used to construct the Hamiltonian projected into the subspace (see eq 3). ${Ĥ}_{S}$ is diagonalized, producing an initial approximation to the target eigenstate ⟨ψ| , from which the first guess for $n_{p\sigma }=\left\langle \psi \left|{â}_{p\sigma }^{†}{â}_{p\sigma }\right|\psi \right\rangle$ .

2. Self-consistent iterations:
  (a) The electronic configurations in X̃ with the wrong particle number and *z*-component of the spin are modified to be in the right symmetry sector by the configuration recovery procedure. Consider the bitstring **x** with *N*_σ_^**x**^ electrons and the target value for the number of σ-electrons is given by *N*_σ_. If *N*_σ_^**x**^>(<)*N*_σ_, |*N*_σ_^**x**^ – *N*_σ_| of the ones (zeros) in **x** are flipped. The probability of each bit **x**_*p*σ_ of being flipped is proportional to the difference between the value of the bit and the average orbital occupancy |**x**_*p*σ_ – *n*_*p*σ_|. This produces the set of recovered configurations ${\mathrm{X̃}}_{R}$ .
  (b) The set of recovered bitstrings is subsampled into *K* batches of configurations $S_{k}$ for *k* = 1, ..., *K*.
  (c) The corresponding Hamiltonians obtained from projecting Ĥ into the $S_{k}$ subspaces ( ${Ĥ}_{S_{k}}$ ) are diagonalized in parallel, obtaining target eigenstates $|\psi^{S_{k}}\rangle$ .
  (d) The average orbital occupancy is computed: $n_{p\sigma }=\frac{1}{K}=\sum_{1\leq k\leq K}\left\langle \psi^{S_{k}}\left|{â}_{p\sigma }^{†}{â}_{p\sigma }\right|\psi^{S_{k}}\right\rangle$ .
  (e) If the stopping criterion is not met, then the new and refined value for *n*_*p*σ_ is sent back to step 2­(a), and steps 2­(a) though 2­(c) are repeated. If the stopping criterion is met, the SQD calculation is concluded. In this study, the stopping criterion is ten recovery iterations.

**Figure 2:**
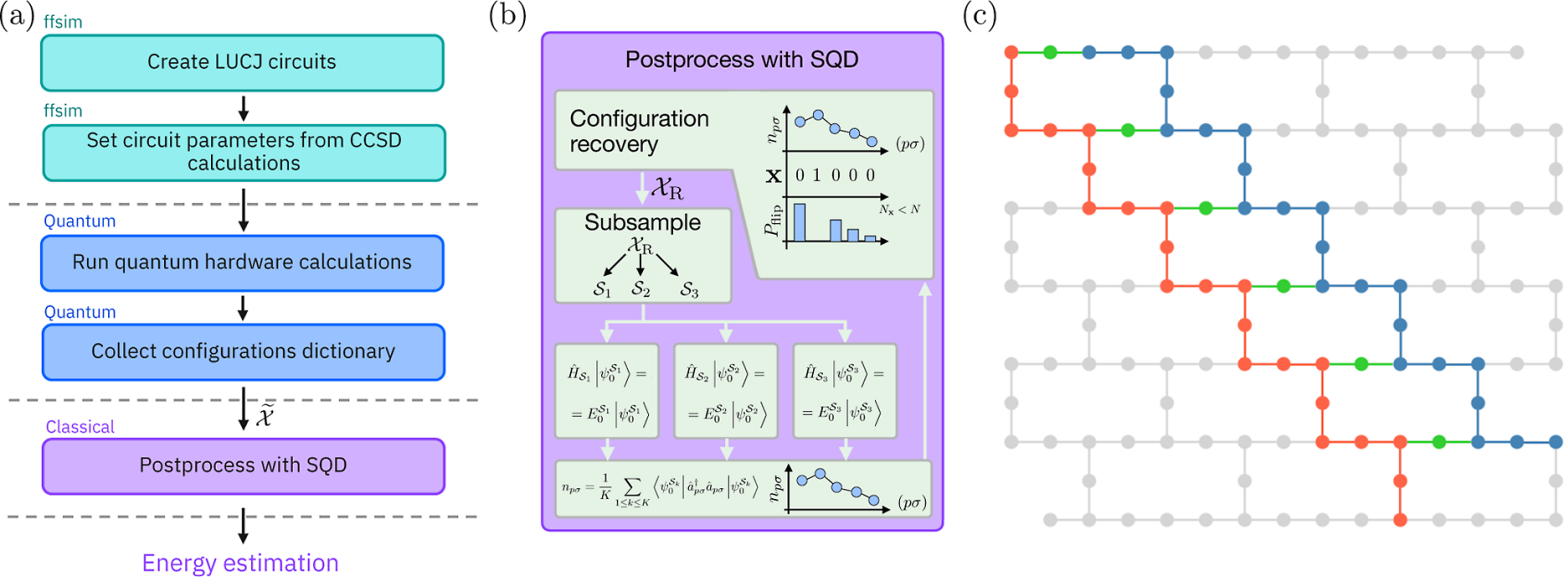
(a) Schematic representation of the workflow that is used in this work to obtain the ground-state energies of methylene. (b) SQD workflow. The set of measurement outcomes from the device X̃ is passed as the input to the configuration recovery subroutine, which restores the correct particle number on bitstrings affected by noise. The recovery step flips bits according to the distance between the bit value and the corresponding orbital occupancy. The resulting set of electronic configurations is subsampled with probabilities following the observation frequencies of each bitstring, forming batches $S_{k}$ . The batches $S_{k}$ define the subspaces that the many-electron Hamiltonian is projected into and diagonalized, producing a collection of approximate ground states. The average orbital occupancy is computed from the approximated eigenstates and sent back into the recovery step. (c) Qubit layouts of Local Unitary Cluster Jastrow (LUCJ) circuits executed in this work for (6e, 23*o*) methylene simulations using 52 qubits on *ibm_nazca*. Qubits used to encode the occupation numbers of α (β) spin-orbitals are shown in red (blue). Auxiliary qubits that mediate the density–density interactions among orbitals of opposite spin are marked in green. Qubit layout was selected to maximize the number of ancilla qubits used to connect spin-up and spin-down orbitals.

*K* batches of samples are considered in order to obtain better statistical convergence in the sampling of configurations, thus producing *K* approximations to the ground state. In this study, we take *K* = 20 for the triplet and *K* = 10 for the singlet.

#### 2.1.3 Total Spin Symmetry

The value of the total spin $S^{2}=\left\langle \psi \left|{Ŝ}^{2}\right|\psi \right\rangle$ is another conserved quantity in the many-electron problem. The conservation of this quantity can be improved by constructing S from the Cartesian product of all spin-up and spin-down configurations in each batch, as detailed in ref 9 Additionally, the addition of a soft constraint in the eigenstate solver improves the adherence to a fixed total spin sector of the Hilbert space

5 $$\left({Ĥ}_{S}+\lambda \left[{Ŝ}^{2}-s\left(s+1\right)\right]\right)|\psi \rangle =E|\psi \rangle$$

where λ is a Lagrange multiplier, whose value is set to λ = 0.2. The specific value used in this work is found by sweeping different values of λ from λ = 0 until the approximate eigenstates produced by SQD yield the desired value of $\left\langle {Ŝ}^{2}\right\rangle$ . The role of the soft constraint is to penalize contributions from the wrong total spin symmetry sector in the construction of the target eigenstates.

In this work, we focus on the calculation of the ground and first excited states of the CH_2_ molecule. In contrast to the recent approach experimentally demonstrated in ref 16 for obtaining excited states, the two eigenstates of interest reside in different U(1) symmetry sectors. The number of spin-up and spin-down electrons is the same in the singlet, while the triplet is obtained by flipping the spin of one of the electrons, resulting in different numbers of spin-up and spin-down electrons. This observation can be exploited to cast the first excited-state calculation as a ground-state calculation in a different symmetry sector.

#### 2.1.4 Orbital Optimization

Orbital optimization is used as a post-SQD step to improve the accuracy of the results. The orbitals are optimized using gradient descent with the ADAM^53^ optimizer, following the produce in ref 54. Some points in the dissociation curve, where lower accuracy was initially reached, required warm starting of the orbital optimization parameters from adjacent points in the dissociation curve that showed good accuracy. The SQD method and orbital optimization were implemented using the qiskit-addon-sqd^55^ python package.

#### 2.1.5 Local Unitary Cluster Jastrow Ansatz

We prepared our wavefunction guesses, used to approximate the ground state, from a truncated version of the LUCJ ansatz.^56^ The corresponding LUCJ circuit was implemented with parameters derived from efficient decomposition of the *T*_2_ amplitudes computed from classical CCSD calculations. A potential decomposition of the 4-index cluster operator into a 2-index operator is achieved with cluster Jastrow ansatz via change in one particle basis. With an efficient parametrization for *T*_2_ amplitudes of generalized qUCCSD, we obtain ${ê}^{\mathrm{T̂}-{\mathrm{T̂}}^{†}}=\prod_{l}e^{{\mathrm{K̂}}^{\left(l\right)}}e^{{Ĵ}^{\left(l\right)}}e^{-{\mathrm{K̂}}^{\left(l\right)}}$ , where *l* is the layer index, and ${\mathrm{K̂}}^{\left(l\right)}$ and ${Ĵ}^{\left(l\right)}$ are one- and two-body operators, respectively

6 K̂(l)=∑pq,σKpq(l)âpσ†âqσĴ(l)=∑pq,στJpq,στ(l)n̂pσn̂qτ

LUCJ is considered an efficient variational quantum eigensolver ansatz capable of reproducing the generalized Unitary Coupled Cluster ansatz for a sufficiently large number of layers. Utilizing the Jordan-Wigner encoding, this ansatz can be realized on a quantum circuit without the Trotter approximation. Each $e^{{\mathrm{K̂}}^{\left(l\right)}}$ can be exactly implemented by a Bogolyubov circuit,^57^ i.e., a brickwork circuit of Givens rotations. Furthermore, each $e^{{Ĵ}^{\left(l\right)}}$ can also be implemented exactly as it involves the exponentiation of commuting Pauli strings comprising only identity and *Z* operations. This ansatz has been demonstrated to yield promising accuracy with reduced circuit depths compared to qUCCSD.^56,58^

In their study, Motta et al.^12^ incorporated additional localization assumptions inspired by the Hubbard model on the *J*_*pq*,σ*τ*_^(*l*)^ “connectivity”, zeroing components of the tensor whose qubits are not adjacent. This integration results in a reduction in quantum computational resources and the ability to customize the approach for various hardware topologies. The depth for each $e^{{\mathrm{K̂}}^{\left(l\right)}}$ component is $O\left(N_{q}\right)$ , where *N*_*q*_ is the number of qubits. The depth for each $e^{{Ĵ}^{\left(l\right)}}$ component, with the locality assumptions is O(1) .

In this study the circuit parameters are obtained from the *t*_1_ and *t*_2_ coefficients from CCSD calculations, as described in ref 9. Two layers of the LUCJ analogue were used. Quantum circuits were constructed using the ffsim^59^ software library, which enabled efficient simulation of Fermionic quantum circuits and formed the foundation for steps 1 and 2 of the workflow in this study (Figure 2a). It has been empirically observed that the parameters derived from CCSD yield good choices to run SQD experiments in noisy quantum processors.^9,13,16,19,60^

### 2.2 Quantum Hardware Calculations

Quantum hardware calculations were conducted on *ibm_nazca*. After transpiling the circuits with the LUCJ ansatz for the *ibm_nazca* backend, we achieved a circuit depth of 1663, with a total of 3324 2-qubit gates. We submitted 40 jobs with various configurations corresponding to different bond distances along the dissociation profile for both the triplet and singlet states. Jobs consisted of 100,000 measurements (shots) for each circuit. Dynamical decoupling^61–63^ and gate twirling^64^ error mitigation techniques were employed to reduce noise originating from quantum gates. The qubit layout is depicted in Figure 2b. When mapped using the Jordan–Wigner transformation, this system was represented by 46 qubits—23 for α and 23 for β spin-orbitals. Six additional qubits served as auxiliary qubits to mediate the density–density interactions between the spin-up and spin-down orbitals.

The LUCJ circuits were sampled in the computational basis to obtain a configuration dictionary containing bit-strings $x\in {\left\{0,1\right\}}^{2N_{orb}}$ that represent electronic configurations (Slater determinants) distributed according to *p̂*(x). As shown in Figure 2a, the collected configuration dictionary is then used in the SQD workflow.

### 2.3 Classical Calculations

Classical calculations were conducted using the open-source Python quantum chemistry package PySCF^46,47^ to establish a reference for our quantum methods and to determine circuit parameters. The geometries for the CH_2_ singlet and triplet were obtained from experimentally determined data (as listed in the NIST CCCBDB database) and used in calculations without any further geometry optimization.^32,33,65^ As shown in Figure 3, at equilibrium, the energy gap between the triplet and singlet states on the cc-pVDZ basis is approximately 18 mE_h_ using SCI and 24 mE_h_ using CCSD. These values are 3.5 and 9.5 mE_h_, respectively, from the experimental singlet-triplet gap. SCI, being the closest classical counterpart to SQD, is chosen as the method of reference to assess the accuracy of the quantum experiments.

**Figure 3:**
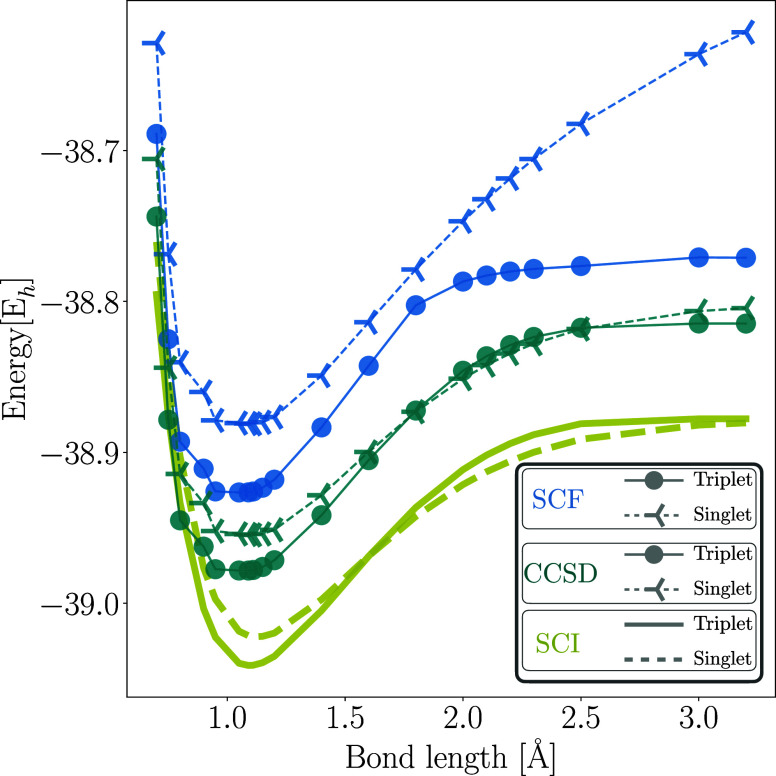
Classical calculations for CH_2_ singlet and triplet dissociation profiles, performed using the cc-pVDZ basis set. The methods included self-consistent field (SCF), coupled cluster singles and doubles (CCSD), and SCI with the frozen core approximation.

The breaking of one of the C–H bonds presents a number of physically relevant features (Figure 3). First, at equilibrium, the triplet is the ground state, while the singlet is an electronically excited state. Upon increasing the bond length, the roles of the singlet and triplet are exchanged in a level crossing, leading to a phase transition of the first order in the ground-state wavefunction. In the large bond length limit, the singlet and triplet energies meet again. This last feature is easily explained by realizing that the electron in the isolated H atom is free to be oriented in any direction along its quantization axis. SCI accurately described these features, while CCSD failed to do so.

## 3 Results and Discussion

All SQD and SCI calculations along the dissociation of methylene in the following section use a subspace dimension of at most 425,104 determinants for the singlet and 241,955 determinants for the triplet calculations.

### 3.1 Energetics and Singlet-Triplet Gap

We first assessed the accuracy of the SQD method in comparison to the SCI and CCSD methods alongside relevant experimental values. As illustrated in Figure 4a,b, the SQD estimates for the triplet energy are in good agreement with those obtained with SCI, with energy differences along the dissociation curve ranging from 1 to 28 mE_h_ and an average difference of approximately 7 mE_h_ in the equilibrium region. Discontinuities and a deterioration of accuracy were observed in the far-dissociation region, particularly between 2.00 and 2.50 Å (Figure 4a,b), which can be attributed to the strongly correlated character of the triplet in that region of the dissociation curve. The SQD results for the CH_2_ singlet, on the other hand, remained within 1–4 mE_h_ of the SCI reference values (Figure 4a,b), demonstrating a remarkable level of agreement across all bond lengths. As shown in panel (b) in Figure 4, the spin contamination in the SQD wavefunctions is minimal, thanks to the penalty term added to the eigenvalue problem (eq 5).

**Figure 4:**
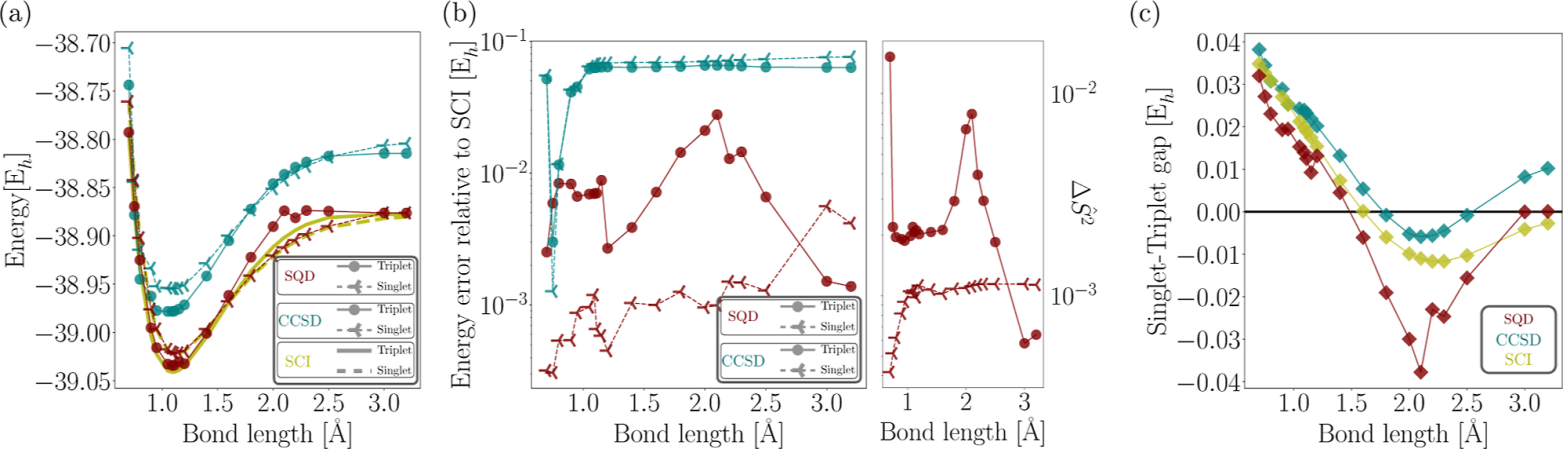
(a) CH_2_ singlet and triplet dissociation energies along the potential energy surface (PES) for bond lengths ranging from 0.75 Å to 3.20 Å, calculated using SQD, CCSD, and SCI. (b) Energy error of SQD and CCSD relative to SCI and spin contamination Δ*S* ^2^ in the SQD wave functions. (c) Singlet-triplet gap as a function of C–H bond length for SQD, CCSD, and SCI calculations.

Figure 4c compares the singlet-triplet energy gap along the C–H bond dissociation pathway, as calculated using the SCI, CCSD, and SQD methods. All three methods predict singlet-triplet gaps at the equilibrium geometry that are in close agreement with each other. At this point, SQD predicts a singlet-triplet energy gap of 19 mE_h_, which closely matches the gap (18 mE_h_) predicted by SCI with the cc-pVDZ basis set (Table 1). Although minor discrepancies are predicted for SQD and SCI results, favorable error cancellation in SQD brings their values into alignment. In order for SQD to predict a singlet-triplet gap that aligns more closely with the experimental value (14 mE_h_) than SCI, larger basis sets and, consequently, experiments with larger quantum circuits would be required. In contrast, the CCSD and SCF methods yield singlet-triplet gaps of 24 mE_h_ and 40 mE_h_, respectively, which are even less accurate than those of SQD. These results highlight that SQD is capable of producing energy gaps that are in strong agreement with both experimental benchmarks and SCI predictions, demonstrating robust performance.

**Table 1:** Comparison of Singlet-Triplet Gap Values Obtained from Various Classical and Quantum Computational Methods with Experimental Data

SCI	SQD	CCSD	SCF	Exp
18 mE_h_	19 mE_h_	24 mE_h_	40 mE_h_	14 mE_h_

As the bond dissociates, the singlet-triplet energy gap becomes vanishingly small (Figure 4c), signaling a first-order phase transition in the ground state. This phenomenon is captured by all three methods, albeit at different critical bond lengths. The critical bond length obtained with SQD is in better agreement with the SCI value as compared to CCSD. Importantly, SQD accurately captures the vanishing of the triplet-singlet gap in the large bond-length limit. This behavior can be attributed to the fact that, at extended C–H bond distances, the electron on the dissociated hydrogen atom is effectively unbound and free to align or anti-align with the quantization axis.

### 3.2 Wavefunction Amplitude Analysis

To better understand the challenges associated with triplet state calculations in the bond length range of 2.00–2.50 Å, we studied the accuracy of individual wavefunction amplitudes obtained from the SQD method, using the SCI method as a benchmark for comparison. Figure 5 shows a comparison between the SCI and SQD wavefunction amplitudes ${\left|\psi_{x}\right|}^{2}$ for both the singlet (Figure 5a) and triplet (Figure 5b) states at various points along the dissociation curve. The agreement between SQD and SCI is stronger for larger wavefunction amplitudes and gradually deteriorates in the description of the tails of the wavefunction. Notably, at larger bond lengths, where higher levels of static correlation are more pronounced, the agreement in small wavefunction amplitudes deteriorates compared to those at bond lengths closer to the equilibrium geometry. This observation can be attributed to two key factors. First, at larger bond lengths, the wavefunction amplitudes are less concentrated compared to those near the equilibrium geometry. The second reason is that at bond lengths near the equilibrium geometry, the wavefunction exhibits a strong mean-field character, which diminishes at larger bond lengths. This directly impacts the shape of the reference spin–orbital occupancy used in configuration recovery. Specifically, when the wavefunction is dominated by mean-field effects, the reference occupancy resembles a sharp step function, with average occupancies of all spin-orbitals close to either 0 or 1. Static correlations can cause this step function to become smoother, resulting in some spin-orbitals having average occupancies that deviate from 0 or 1. The recovery of configurations in this study is more effective when the components of *n* are close to these values.

**Figure 5:**
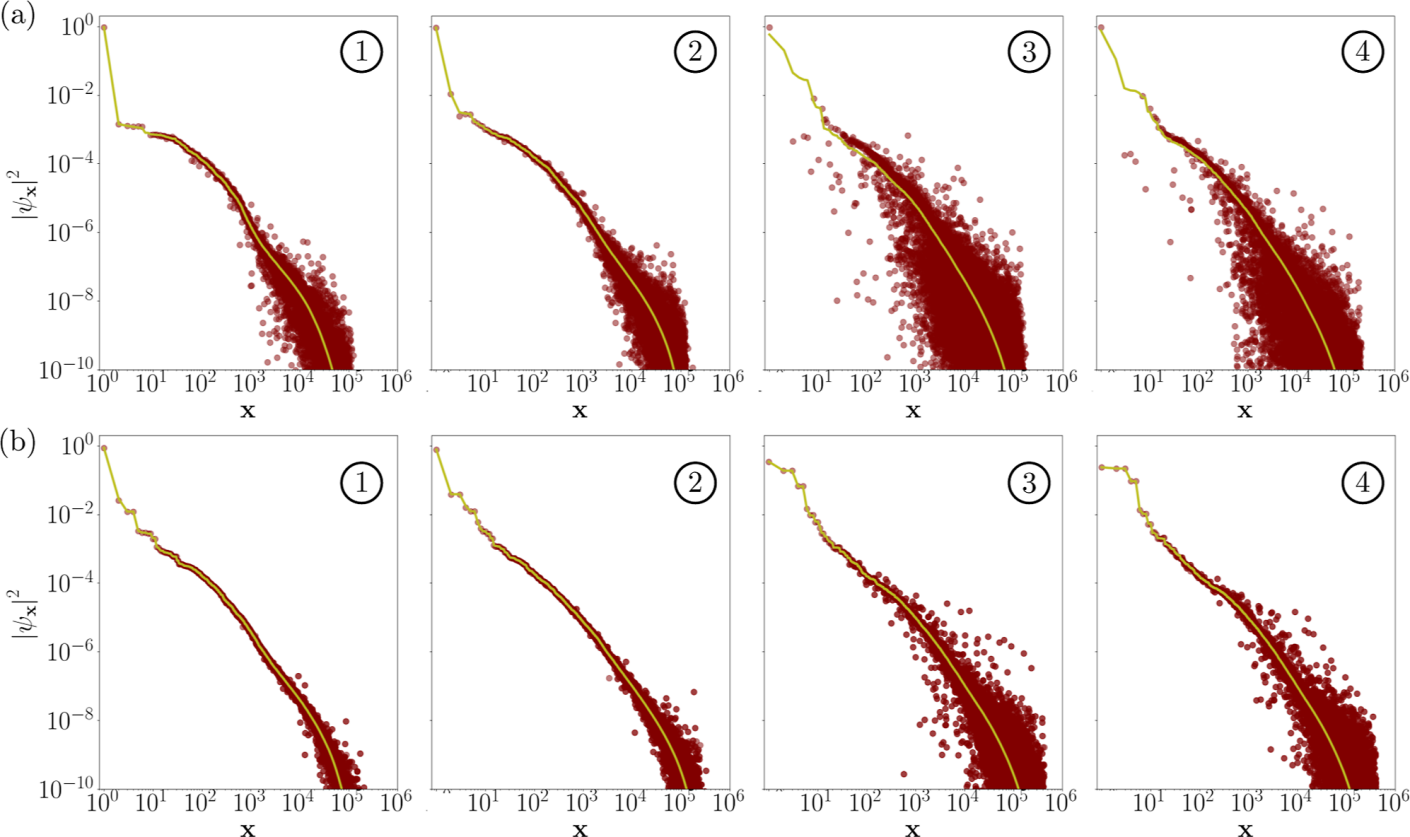
Comparison of the SCI (yellow solid line) and SQD (brown dots) wavefunction amplitudes for all electronic configurations x∈S . The configurations **x** are sorted along the horizontal axis in a descending order of ${\left|\psi_{x}\right|}^{2}$ of the SCI wavefunction. Panels (a) and (b) correspond to the singlet and triplet states, respectively, at bond lengths 1.11 Å → ①; 1.60 Å → ②; 2.30 Å → ③; 2.50 Å → ④.

Multi-reference character is more pronounced for CH_2_ triplet state SQD calculations for bond lengths between 2.00 and 2.50 Å, which contributes to the observed decrease in accuracy compared to the singlet state within the same range. Despite this, SQD produces accurate representations of the ground-state wavefunction, even at the level of individual amplitudes. However, the challenges posed by less concentrated wavefunctions are notable; the accuracy of the SCI solver decreases, and configuration recovery becomes less effective due to the softer profiles of *n*. Overall, this analysis underscores the performance of the SQD while highlighting the specific difficulties encountered in this region.

## 4 Conclusions

In this study, we conducted electronic structure calculations for the two lowest lying states of the CH_2_ molecule using quantum hardware. We specifically focused on the ground-state triplet and the first excited-state singlet employing the SQD method within a quantum-centric simulation framework. This approach integrates quantum and classical calculations, enabling large-scale quantum computations and comprehensive postprocessing of the data obtained from quantum hardware. Importantly, this research represents the first application of the SQD method to an open-shell molecule, particularly the CH_2_ triplet state.

We evaluated the effectiveness of the SQD algorithm for both open- and closed-shell systems using a (6e, 23o) CH_2_ molecular system and conducting quantum hardware calculations on a 52-qubit system. The SQD results for the CH_2_ singlet state demonstrated excellent agreement with SCI results, deviating by only a few mE_h_. In contrast, the results for the triplet state were less consistent with SCI; however, they remained within a few mE_h_ values at equilibrium. The singlet-triplet energy gap showed close agreement with both SCI and experimental results, primarily due to beneficial error cancellation. However, in the triplet dissociation region, SQD struggled to capture the wavefunction character, an issue not observed with the singlet state. A potential extension to SQD involves developing new strategies to diversify the ensemble of bit-strings in the open-shell case, such as constructing compatible subsets of spin-up and spin-down configurations. These approaches could enhance the representation of eigenvector character in open-shell systems and will be investigated in future work.

Overall, this study enhances our understanding of the SQD method for both closed- and open-shell systems, laying the groundwork for future advancements and applications in accurate electronic structure studies using large-scale, noisy quantum computers. In aerospace and defense applications, high-fidelity quantum chemical calculations play a critical role in modeling complex chemical environments. By advancing theoretical approaches, such as those benchmarked with the CH_2_ molecule, these simulations could enhance predictive models that can support the development of innovative sensing and detection technologies. The results in this work indicate that SQD has the potential to enable accurate electronic calculations for larger radical and transient species as well as complex reactions relevant to combustion chemistry. Future work includes continued research on how SQD could expand the scale of what is possible with currently available and future hardware. As lower noise and partially or fully error-corrected hardware becomes available, this technique, or extensions to it, may pave the way for early utility-scale applications and use cases in quantum computing.
